# Root and Leaf-Specific Metabolic Responses of Ryegrass to Arbuscular Mycorrhizal Fungi Under Cadmium Stress

**DOI:** 10.3390/jof12010074

**Published:** 2026-01-19

**Authors:** Dapeng Jin, Lingyu Xin, Panpan Tu, Huiping Song, Yan Zou, Zhiwei Bian, Zhengjun Feng

**Affiliations:** 1 Institute of Resources and Environmental Engineering, Shanxi University, Taiyuan 030006, China; 15735142376@163.com (D.J.); xinlingyu1234@163.com (L.X.); t9270065@163.com (P.T.); songhp@sxu.edu.cn (H.S.); 2 Shanxi Qinghuan Nengchuang Environmental Protection Technology Company Limited, Taiyuan 030006, China; 3 China-Mongolia Belt and Road Joint Laboratory of Mineral Processing Technology, Inner Mongolia Academy of Science and Technology, Hohhot 010018, China

**Keywords:** arbuscular mycorrhizal fungi, cadmium stress, metabolomic profiling, root-leaf metabolic responses

## Abstract

Cadmium (Cd) drastically inhibits plant growth and metabolism, whereas arbuscular mycorrhizal (AM) fungi can enhance plant Cd tolerance through metabolic regulation. To clarify tissue-specific responses, we conducted a pot experiment combined with GC-MS to examine how AM fungi influence root and leaf metabolism of ryegrass (*Lolium perenne* L.) under different Cd levels. Root and leaf metabolomes diverged substantially in composition and function. In total, 83 metabolites were identified in roots, mainly phenolics, amines, and sugars associated with carbon–nitrogen metabolism and stress-defense pathways, whereas 75 metabolites were identified in leaves, largely related to photosynthetic metabolism. Roots were more sensitive to Cd, showing significant metabolic alterations at Cd ≥ 5 mg·kg^−1^, including disruption of galactose metabolism, while leaves exhibited notable changes only at Cd ≥ 100 mg·kg^−1^, with suppression of citrate, L-aspartate, and starch and sucrose metabolism. AM fungi modulated plant metabolism more strongly under Cd stress. Specifically, AM fungi restored Cd-suppressed galactose and glyoxylate/dicarboxylate metabolism in roots, enhanced starch and sucrose metabolism and amino acid pathways in leaves, and increased stress-related amino acids and organic acids in both tissues. Overall, AM fungi substantially alleviated Cd-induced metabolic inhibition, particularly at Cd ≥ 50 mg·kg^−1^, providing mechanistic insight into AM-enhanced Cd tolerance and supporting the application of AM symbiosis in remediation of Cd-contaminated soils.

## 1. Introduction

Cadmium (Cd) is a highly toxic heavy metal with strong bioaccumulation and high mobility, posing severe ecological and human health risks [1]. In China, heavy-metal pollution in agricultural soils remains a critical environmental challenge [2,3]. According to the Bulletin of National Soil Pollution Survey [4,5], Cd displays the highest exceedance rate (7.0%) among all inorganic soil contaminants nationwide [6]. It also threatens human health via food-chain transfer, leading to impaired calcium metabolism and renal dysfunction [7]. Therefore, arbuscular mycorrhizal (AM) fungi have received increasing attention as a promising biological strategy for mitigating Cd pollution [8]. Forming symbioses with more than 80% of terrestrial plant species [9,10], AM fungi can alleviate heavy-metal stress by broadening the soil–root interface, adjusting rhizosphere pH, and reorganizing root structure [11,12]. At the same time, AM fungi can increase plant uptake of key nutrients such as nitrogen, phosphorus, and potassium [13].

Plant metabolomics as a subset of systems biology can be used to profile metabolite dynamics in response to the stress condition at a high-resolution scale, creating indispensable molecular understanding of plant adaptive response [14]. Prior studies have demonstrated that plants can cope with the stress of Cd by altering the concentrations of amino acids, organic acids, phenolic compounds and other minor metabolites; such as organic acids reduce Cd bioavailability through chelation, while phenolic compounds alleviate oxidative damage by scavenging reactive oxygen species (ROS) [15]. Nevertheless, these studies currently leave two broad gaps in metabolomic studies of AM symbioses to Cd stress: (i) a predominant focus on single-organ responses, particularly in leaves, with insufficient attention to coordinated root-leaf metabolic regulation; and (ii) an incomplete mechanistic understanding of how AM fungi remodel host metabolic pathways to enhance Cd tolerance.

As integrated belowground-aboveground organisms, plants rely on roots for nutrient acquisition and also for long-distance signaling. Metabolic shifts in roots can modulate leaf metabolism through mineral transport and hormone signaling pathways. Distinct inherent metabolic profiles between roots and leaves have been widely reported [16]. For instance, non-targeted metabolomics of *Astragalus membranaceus* detected 2190 compounds across roots, stems, and leaves, with bioactive metabolites preferentially accumulating in roots despite higher total metabolite abundance in aerial tissues [17]. Similarly, organ-specific N-containing metabolites have been identified in *Catharanthus roseus*, detecting 15 root-specific and 26 leaf-specific metabolites [18] and LC-MS analysis of blackberry (*Rubus fruticosus*) revealed striking metabolic divergence between roots and leaves [19].

Recent studies on heavy-metal stress suggest a transition from root-centric analyses to root-leaf integrated metabolic models. Stresses on Cd and Zn in maize with *Claroideoglomus claroideum* caused a wide shift in primary and secondary metabolism and lignin composition, and AM fungi fixed soil heavy-metal ions and inhibited their uptake into plant tissues [20]. Wang et al. further revealed organ-specific metabolic strategies in *Solanum nigrum* under Cd stress [21]. Roots also showed increased levels of glycolysis intermediates and higher TCA cycle activity to sustain energy-demanding detoxification (e.g., sequestration via ABC transporters). Conversely, leaves activated glutathione and amino acid metabolism, which increased ROS scavenging, osmotic regulation, and Cd chelation, forming an “antioxidation and repair” metabolic strategy.

AM fungi are also known to enhance plant resilience to diverse abiotic stresses through the regulation of primary and secondary metabolism. Under low temperature stress, AM inoculation in cucumber caused a significant boost in the lignin, phenolics and flavonoid accumulation core constituents of the antioxidative system, and thus the cold tolerance was enhanced [22]. A metabolomic analysis conducted on *Salvia miltiorrhiza* when subjected to the drought stress condition has shown marked alteration in amino acid, sugar, and polyol metabolic pathways, and provided an indication of a difference in the effects brought by drought stress on shikimate-mediated polyphenol biosynthesis when the organism was subjected to light versus subjected to air-drying [23]. In addition, AM fungi mitigate Cd toxicity in maize that modulates Cd subcellular localization and chemical speciation [24].

Based on these findings, the current experiment uses perennial ryegrass (*Lolium perenne* L.) as the host plant that is highly resistant to stress [25] and *Rhizophagus irregularis* as the inoculum of AM. Through a pot experiment conducted with the help of a gas chromatography-mass spectrometry (GC-MS) metabolomics [26], we methodically study the action of AM fungi on plant growth, Cd uptake and translocation, and orchestrated root-leaf metabolic responses to Cd stress. This paper can explain how AM fungi contribute to plant tolerance to Cd in terms of the theoretical basis by the way of combined analyses of root and leaf metabolomes in the context of AM symbiosis, thus providing a theoretical framework on the application of AM symbioses in the ecological remediation of Cd-contaminated soils.

## 2. Materials and Methods

### 2.1. Preparation of Cd-Contaminated Soil

Soil was collected from Wuxiang County, Shanxi Province, China (36°50′ N, 112°51′ E). The soil had organic matter of 32.33 g·kg^−1^, total nitrogen of 0.70 g·kg^−1^, available phosphorus of 15.61 mg·kg^−1^ and the pH was 8.57. The initial Cd concentration was 0.575 mg·kg^−1^. The soil was air-dried, ground and sieved through a 2 mm mesh, and sterilized by autoclaving at 121 °C and 0.1 MPa for 2 h. Cadmium was added as a CdCl_2_ solution to achieve target concentrations of 0, 5, 50, 100, and 200 mg·kg^−1^. Ultrapure water was added occasionally to promote complete homogenization, and the amended soils were equilibrated for one month until Cd adsorption stabilized.

### 2.2. Experimental Design

Perennial ryegrass (*Lolium perenne* L.) was used as the test plant and seeds were sourced from Zhongjiang Seed Industry Co., Ltd. (Nanjing, China). AM fungal inoculum was composed of *Rhizophagus irregularis* powder (Symbiom, Sázava, Czech Republic). Five Cd levels (0, 5, 50, 100, and 200 mg·kg^−1^) were established, each with two treatments: no AM inoculation control (NM), and AM inoculation (AM). Each treatment included five replicates (50 pots in total).

Pot experiments were performed in sterile plastic containers containing 2300 g of soil (12.5 × 17 × 16 cm). Seeds were surface-sterilized with 10% H_2_O_2_ for 10 min. In each pot, 1.50 g of seeds and 0.10 g of AM inoculum were mixed and planted at two thirds of the pot depth; NM treatments received the same amount of autoclaved inoculum. The seeds were covered with 2 cm of soil. Greenhouse conditions were maintained at a 14 h light period, approximately 25 °C, and regular irrigation with ultrapure water.

### 2.3. Sample Collection and Measurements of Growth and Physiological Parameters

Plants were harvested after 60 d. Plant height, shoot fresh weight, and root biomass were recorded. Root mycorrhizal colonization was assessed using trypan blue staining [27]. Chlorophyll content was measured using a portable SPAD meter. Soluble sugars, malondialdehyde (MDA), and antioxidant enzyme activities—including total superoxide dismutase (T-SOD), peroxidase (POD), and catalase (CAT)—were quantified using commercial kits (Nanjing Jiancheng Bioengineering Institute, Nanjing, China).

Plant tissues were digested using a mixed acid solution (HNO_3_:H_2_O_2_ = 4:1, *v*/*v*), and Cd concentrations in roots and shoots were determined using ICP-MS. The bioconcentration factor (BCF) was calculated as the ratio of Cd concentration in plant tissues to that in soil, and the translocation factor (TF) as the ratio of shoot Cd concentration to root Cd concentration. Fresh roots and leaves were freeze-dried for 72 h and ground to a fine powder for metabolomic analysis.

### 2.4. Metabolomic Analysis of Plant Tissues

Metabolomic profiling was performed on fresh roots and leaves from the ten treatments, with three biological replicates each. GC-MS data were processed using MassHunter Qualitative Analysis B.07.00, and metabolites were identified via the NIST14 mass spectral library. Normalized data were analyzed using the MetWare platform (https://cloud.metware.cn, accessed date: 9 July 2025) for principal component analysis (PCA), differential metabolite screening, and heatmap visualization. MetaboAnalyst 6.0 was used to perform pathway enrichment and statistical analysis (https://www.metaboanalyst.ca/, accessed date: 9 July 2025).

### 2.5. Statistical Analysis

Two-way ANOVA was conducted to determine the influence of AM inoculation and Cd exposure on morphological characteristics of plants, mycorrhizal colonization, chlorophyll content, soluble sugars, antioxidant enzyme activities (T-SOD, POD, CAT), and MDA levels. Post hoc analysis was performed using Duncan’s multiple range test. Statistical analyses were conducted using IBM SPSS Statistics 23.0 (IBM Corp., Armonk, NY, USA). Data are presented as mean ± standard deviation (*n* = 5), and differences were considered statistically significant at *p* < 0.05.

## 3. Results

### 3.1. Growth and Cadmium Accumulation in Ryegrass Under AM Fungal Inoculation

**Figure 1:**
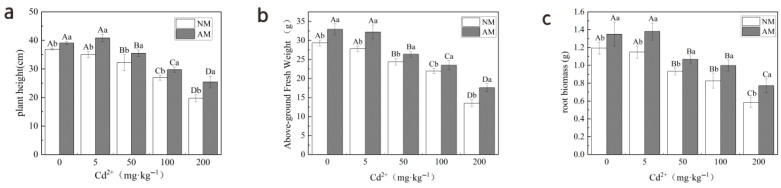
Changes in growth parameters of ryegrass under different treatments: (**a**) plant height, (**b**) shoot fresh weight, and (**c**) root biomass. NM represents the non-mycorrhizal (non-inoculated) treatment, and AM represents the arbuscular mycorrhizal (AM) fungal inoculation treatment. Data are presented as mean ± standard deviation (mean ± SD). Different lowercase letters indicate significant differences between NM and AM treatments, while different uppercase letters indicate significant differences among Cd concentration treatments.

Figure 1 indicates a progressive reduction in plant height, above-ground fresh weight, and root biomass of ryegrass without AM fungal inoculation (NM group) with increasing Cd concentration, compared to the control group (0 mg·kg^−1^). The effect was not significant at low levels (≤5 mg·kg^−1^), but was significant at high levels (≥50 mg·kg^−1^). In contrast, ryegrass inoculated with AM fungi (AM group) exhibited significant growth advantages across all Cd concentrations. Plant height increased by 5.85% to 29.27%, above-ground fresh weight increased by 7.04% to 30.14%, and root biomass increased by 13.26% to 32.65%, with the most notable effect observed at the highest Cd concentration (200 mg·kg^−1^). These results indicate that AM fungi effectively mitigate the inhibitory effects of Cd on ryegrass growth.

With increasing Cd concentration, the Cd content in both roots and leaves of ryegrass significantly increased, with root Cd concentrations being much higher than those in leaves. AM fungal inoculation significantly enhanced Cd accumulation in roots. However, as Cd concentration increased, the rate of increase in root Cd accumulation due to AM fungi gradually decreased. As shown in Table 1, the root Cd enrichment coefficient initially increased and then decreased with rising Cd concentrations, while the leaf Cd enrichment coefficient consistently increased. The root enrichment coefficient (2.66–13.75) was always higher than the leaf enrichment coefficient (0.37–0.86), and in all treatments, the root enrichment coefficient was greater than 1, indicating that Cd primarily accumulated in roots. AM fungal inoculation significantly enhanced the root and leaf enrichment coefficients (increased by 35.0–79.3%), thereby improving ryegrass’s ability to absorb Cd. Furthermore, AM fungi significantly reduced the root-to-leaf transport coefficient by 8.7% to 33.3%, indicating enhanced Cd fixation in roots and reduced Cd transport to the above-ground parts of the plant.

### 3.2. Mycorrhizal Inoculation Rate

Table 2 shows the impact of AM fungal inoculation on ryegrass mycorrhizal colonization rates under Cd stress. With increasing Cd concentration, the mycorrhizal infection rate showed a decreasing trend. In the absence of Cd stress, the colonization rate was highest at 56.71%. At a Cd concentration of 200 mg·kg^−1^, the colonization rate dropped to approximately half (28.91%). There was a lack of significant differences in mycorrhizal colonization rate at low Cd concentrations (≤5 mg·kg^−1^). However, beyond a certain Cd concentration (≥50 mg·kg^−1^), mycorrhizal colonization was significantly inhibited, indicating that low Cd levels did not influence colonization, while higher Cd concentrations significantly inhibited AM fungal colonization.

### 3.3. Physiological Responses to AM Fungi Inoculation

**Figure 2:**
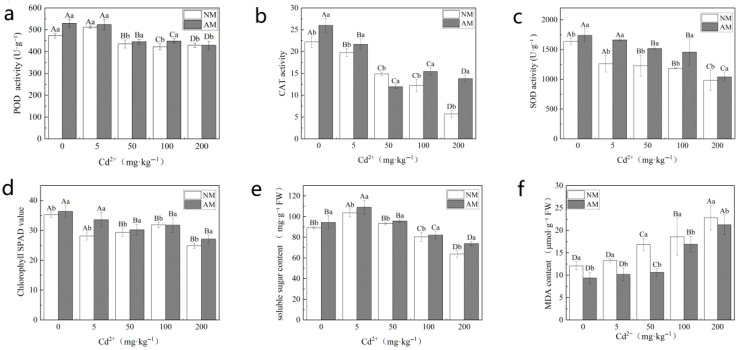
Changes in physiological parameters of ryegrass under different treatments: (**a**) peroxidase (POD) activity; (**b**) catalase (CAT) activity; (**c**) superoxide dismutase (SOD) activity; (**d**) chlorophyll content; (**e**) soluble sugar content; and (**f**) malondialdehyde (MDA) content. Note: NM denotes the non-mycorrhizal (non-inoculated) treatment, whereas AM denotes the arbuscular mycorrhizal (AM) fungal inoculation treatment. Data are presented as mean ± SD (*n* = 5). Different letters indicate significant differences among treatments at *p* < 0.05. Lowercase letters represent differences between NM and AM treatments, and uppercase letters represent differences among Cd concentration treatments.

As shown in Figure 2, cadmium (Cd) stress significantly affected multiple physiological parameters of perennial ryegrass, whereas arbuscular mycorrhizal (AM) fungal inoculation markedly alleviated Cd-induced physiological disturbances. In non-mycorrhizal (NM) plants, antioxidant enzyme activities (POD, CAT, and SOD) increased moderately at low Cd levels (≤50 mg·kg^−1^) but declined under high Cd stress (≥100 mg·kg^−1^) (Figure 2a–c). In contrast, AM inoculation significantly enhanced POD, CAT, and SOD activities under Cd stress, with consistently higher levels than NM plants at Cd concentrations ≥ 50 mg·kg^−1^ (*p* < 0.05).

Cd stress significantly reduced chlorophyll content in NM plants, particularly at Cd concentrations ≥ 100 mg·kg^−1^ (Figure 2d). AM-inoculated plants maintained significantly higher chlorophyll levels across all Cd treatments, with increases of approximately 12–16% at 200 mg·kg^−1^ Cd compared with NM plants (*p* < 0.05). Soluble sugar content decreased progressively with increasing Cd concentration in NM plants (Figure 2e), whereas AM inoculation significantly increased soluble sugar levels under moderate and high Cd stress (≥50 mg·kg^−1^), with enhancements of approximately 6–16% relative to NM plants (*p* < 0.05).

Malondialdehyde (MDA) content increased significantly with rising Cd levels in NM plants (Figure 2f). AM fungal inoculation significantly reduced MDA accumulation under Cd stress, particularly at Cd concentrations ≥ 50 mg·kg^−1^, resulting in an 18–25% reduction at 200 mg·kg^−1^ Cd compared with NM plants (*p* < 0.05). Collectively, these results demonstrate that AM fungi enhance ryegrass tolerance to Cd stress by strengthening antioxidant defense, maintaining photosynthetic capacity, and improving osmotic regulation, with protective effects becoming more pronounced as Cd stress intensity increased.

### 3.4. Metabolomics Analysis Results

#### 3.4.1. Identification and Classification of Metabolites in Roots and Leaves

**Figure 3:**
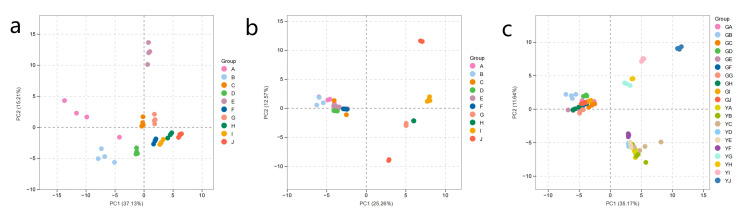
Principal component analysis (PCA) of root and leaf metabolites of ryegrass inoculated with AM fungi under Cd stress. (**a**) PCA of root metabolites; (**b**) PCA of leaf metabolites; (**c**) PCA of combined root-leaf metabolites. A, B, C, D, E, F, G, H, I, and J correspond to Cd0, Cd0M, Cd5, Cd5M, Cd50, Cd50M, Cd100, Cd100M, Cd200, and Cd200M, respectively.

Principal component analysis (PCA) was employed to assess metabolic differentiation between roots and leaves of perennial ryegrass under Cd stress and AM fungal inoculation (Figure 3).

For root tissues, PC1 and PC2 accounted for 37.13% and 15.21% of the total variation (52.34% cumulatively). Samples from the control group were clearly separated from all Cd and AMF treatments, indicating that both Cd exposure and AM symbiosis substantially reshaped root metabolic profiles. In leaves, PC1 and PC2 explained 25.26% and 12.57% of the variation (37.83% in total). Treatments with higher Cd levels (≥100 mg·kg^−1^) diverged markedly from low-Cd groups, suggesting that leaf metabolism is more sensitive to high Cd stress.

The integrated PCA of root and leaf samples indicated a strong organ-level distinction: root samples were located on the left side of the *x*-axis, and leaf samples on the right, indicating that the two organs had different metabolic phenotypes. Taken altogether, these findings indicate that Cd and AMF significantly affect root metabolism, and that higher Cd levels have more severe effects on leaf metabolism.

#### 3.4.2. Differential Metabolite Profiles in Roots and Leaves

**Figure 4:**
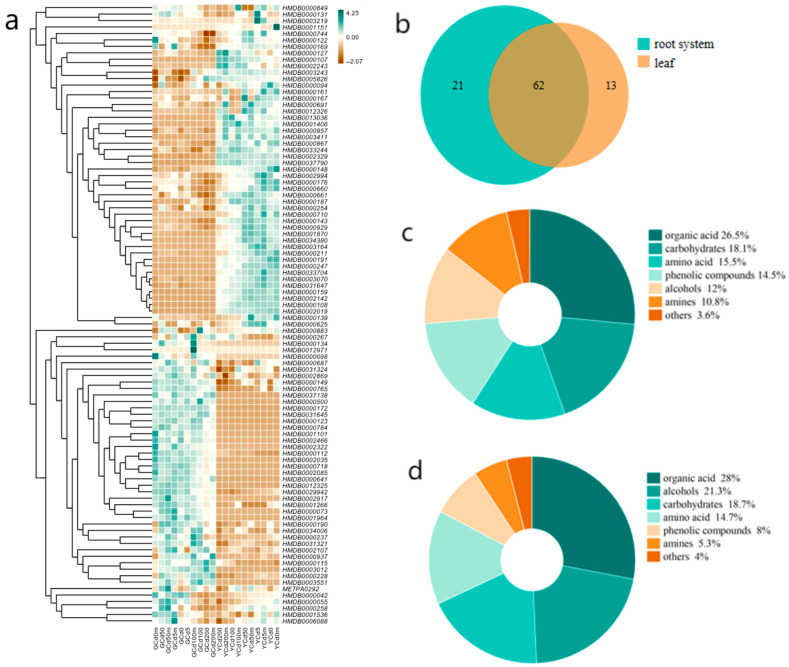
(**a**) Heatmap of metabolite profiles in roots and leaves (G denotes roots, and Y denotes leaves); (**b**) heatmap of metabolites in roots and leaves; (**c**) classification of root metabolites; (**d**) classification of leaf metabolites.

Eighty-three metabolites were identified in roots and 75 in leaves, including amino acids, sugars, and organic acids as primary metabolites, as well as phenolics, polyols, amines, and steroids as secondary metabolites. Figure 4a illustrates variation in metabolite abundance among treatments using a color-coded heatmap (brown indicates the lowest abundance and white indicates the highest). Metabolites exhibited organ-specific accumulation patterns.

As shown in Figure 4b, 62 metabolites were shared between roots and leaves. Twenty-one metabolites, mainly phenolics, amines, and organic acids, were root-specific, while thirteen metabolites—primarily polyols and organic acids—were uniquely detected in leaves. Figure 4c,d further indicate that roots were enriched in sugars, phenolics, and amines, whereas leaves accumulated higher levels of polyols.

#### 3.4.3. Screening of Differential Metabolites in Roots and Leaves of Ryegrass Inoculated with Arbuscular Mycorrhizal Fungi Under Cadmium Stress

##### Screening of Differential Root and Leaf Metabolites of Ryegrass Under Single Cadmium Stress

**Figure 5:**
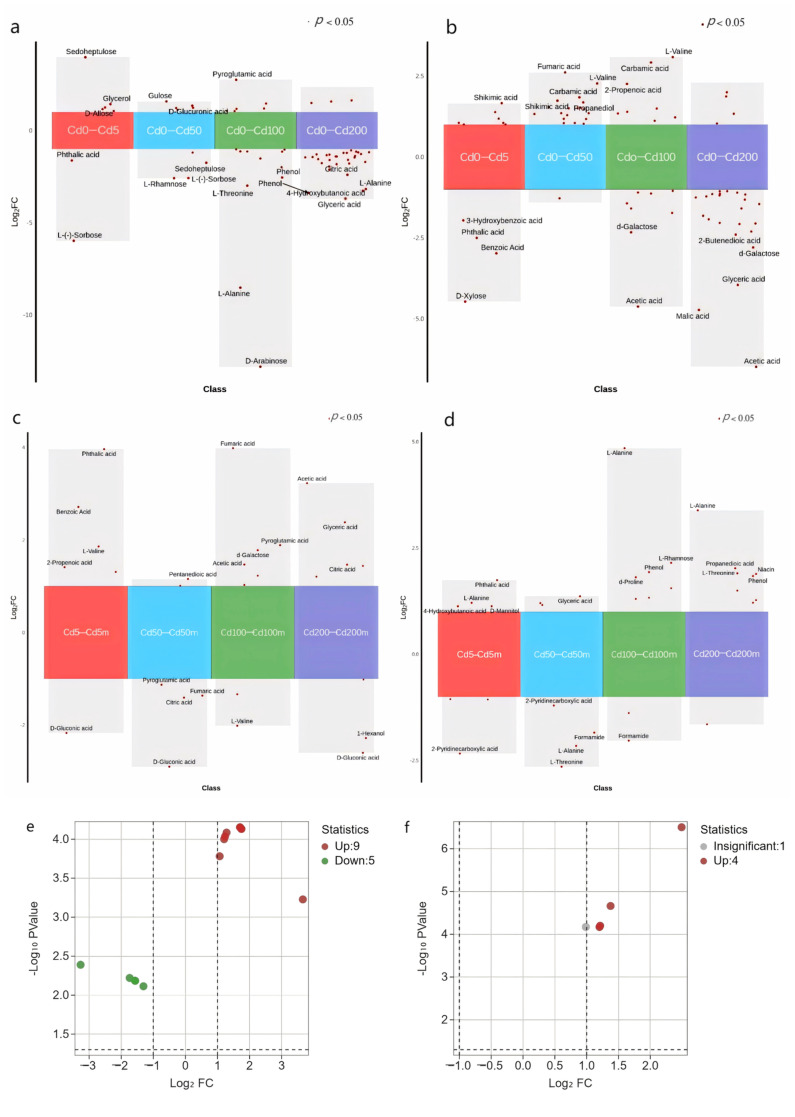
Volcano plots illustrating differential metabolites in ryegrass roots and leaves across treatments. (**a**,**b**) Differential metabolites in roots (**a**) and leaves (**b**) under varying Cd stress; (**c**,**d**) differential metabolites in roots (**c**) and leaves (**d**) of AM-inoculated ryegrass under Cd stress; (**e**,**f**) metabolite changes in roots (**e**) and leaves (**f**) following AM fungal inoculation.

As Cd concentration increased, the number of differential metabolites in roots also increased (Figure 5a), indicating substantial metabolic disturbance. At 50 mg·kg^−1^ Cd, upregulated metabolites were predominant (91.3%), mainly organic acids and phenolics associated with stress resistance.

At 200 mg·kg^−1^ Cd, 75.76% of metabolites were downregulated, especially sugars, organic acids, and phenolics, reflecting inhibited root metabolism under severe Cd toxicity. Based on log_2_(FC) ranking, the top five differential metabolites at 5 mg·kg^−1^ Cd were all upregulated. With higher Cd concentrations, the proportion of downregulated metabolites increased, highlighting the suppressive effect of Cd on metabolic activity and plant growth. Carbamic acid and 2-propenoic acid were consistently elevated across all Cd concentrations. Succinate was significantly upregulated at 5 and 50 mg·kg^−1^, phenol increased at 50–200 mg·kg^−1^, and D-galactose decreased significantly at 100–200 mg·kg^−1^.

In leaves (Figure 5b), a similar concentration-dependent trend emerged. At ≤50 mg·kg^−1^ Cd, few metabolites changed, and ~60% were upregulated (mainly organic acids and sugars). At ≥100 mg·kg^−1^ Cd, the number of differential metabolites increased sharply, with 73.91% and 85.37% downregulated at 100 and 200 mg·kg^−1^. D-allose increased at high Cd levels; L-sorbose, citrate, and L-aspartate declined across all Cd treatments.

Overall, Cd stress significantly affected both root and leaf metabolites.

##### Screening of Differential Root and Leaf Metabolites of Ryegrass Inoculated with Arbuscular Mycorrhizal Fungi

Differential metabolites following AM fungal inoculation were screened using VIP > 1, *t*-test, and *p*-values. Volcano plots (Figure 5e,f) show that AM inoculation induced stronger metabolic changes in roots than in leaves.

In roots, β-sitosterol, D-gluconic acid, aniline, citrate, and lactic acid decreased significantly, while fumarate, cadaverine, L-threonine, D-mannose, glyceric acid, D-glucose, formamide, carbamic acid, and D-xylose were elevated. In leaves, pyroglutamate, ethanolamine, formamide, and D-mannitol significantly increased. These results indicate a stronger metabolic response to AM fungi in roots.

##### Screening of Differential Metabolites in Response to AM Inoculation Under Cd Stress

Under Cd stress, AM fungi increased the abundance of most differential metabolites in roots (Figure 5c), particularly organic acids, sugars, and alcohols. Across Cd concentrations, D-gluconic acid consistently decreased, while acetate increased significantly at 50, 100, and 200 mg·kg^−1^. D-mannose increased at ≤50 mg·kg^−1^ Cd.

In leaves (Figure 5d), AM fungi increased most metabolite levels under Cd stress. Phenol and L-alanine increased at ≥100 mg·kg^−1^ Cd, while D-allose decreased at ≤50 mg·kg^−1^.

Overall, AM fungi enhanced the accumulation of metabolites associated with stress response under Cd exposure.

#### 3.4.4. Metabolic Pathway Analysis

Using the OPLS-DA model with VIP values above 1, together with Student’s *t*-tests and associated *p*-values, we characterized metabolite changes in ryegrass roots and leaves under Cd exposure following AM fungal inoculation. In total, 37 differential metabolites were identified in each tissue type. KEGG annotations mapped these metabolites to 21 metabolic pathways in roots and 25 in leaves. At the significance threshold of *p* ≤ 0.05, seven pathways in roots and ten in leaves showed significant enrichment, while two root pathways and seven leaf pathways exhibited highly significant enrichment at *p* ≤ 0.01.

For roots, enrichment analyses highlighted two major pathways: galactose metabolism and glyoxylate and dicarboxylate metabolism. In leaves, enriched pathways included: (1) galactose metabolism; (2) starch and sucrose metabolism; (3) fructose and mannose metabolism; (4) branched-chain amino acid biosynthesis (valine, leucine, and isoleucine); (5) alanine, aspartate, and glutamate metabolism; (6) ascorbate and aldarate metabolism; and (7) glyoxylate and dicarboxylate metabolism. Functionally, these pathways can be categorized into biosynthetic routes (carbohydrate, amino acid, and fatty acid synthesis) and energy-yielding metabolic processes (primarily carbohydrate catabolism).

**Figure 6:**
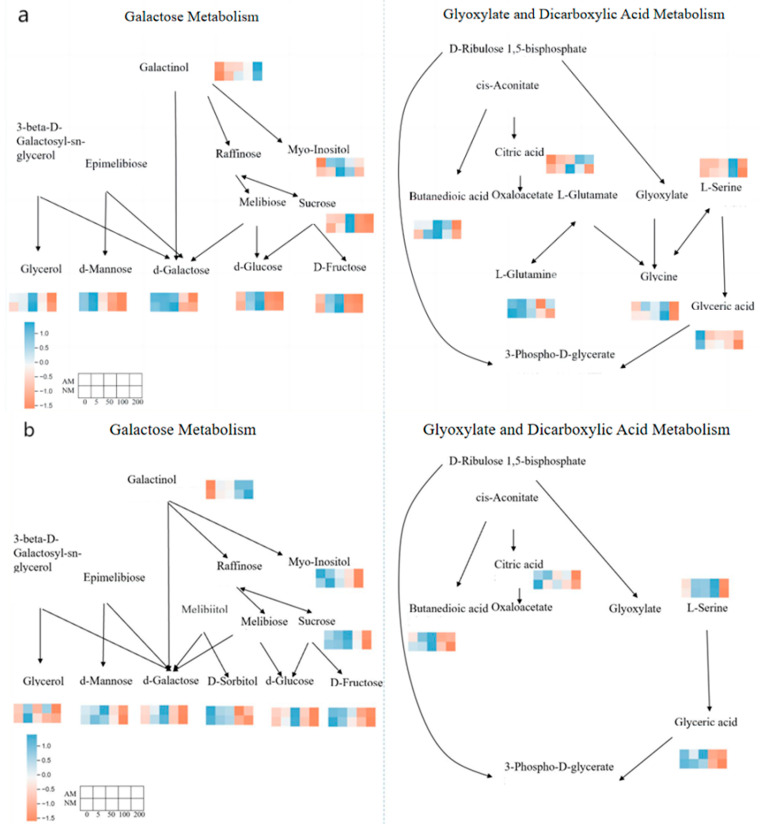
Metabolic pathways of root and leaf metabolites in AM-inoculated ryegrass under Cd stress. (**a**) Metabolic pathways in roots; (**b**) metabolic pathways in leaves.

Because galactose metabolism and glyoxylate/dicarboxylate metabolism were enriched in both tissues, they were examined in greater detail. Carbohydrates act as primary substrates for plant energy production and play pivotal roles in growth, osmotic regulation, and stress defense. Under high Cd stress (Figure 6), glycerol, D-galactose, sucrose, and other metabolites were significantly reduced, indicating a strong inhibition of galactose metabolism. These reductions were reversed by AM fungal inoculation. AM fungi restored core carbohydrate metabolic turnover and supported physiological stability during Cd exposure.

Tightly linked to the TCA cycle, glyoxylate–dicarboxylate metabolism is also sensitive to Cd. Excessive Cd exposure significantly decreased the abundance of core TCA intermediates, such as succinate, citrate, and glycerate, indicating a disturbance of central carbon metabolism. AM fungal inoculation increased the levels of these intermediates, indicating partial restoration of TCA cycle activity. This restoration likely contributes to improved cellular energy balance and enhanced metabolic resilience under Cd stress.

Collectively, these results suggest that AM fungi alleviate Cd-induced metabolic disorders mainly through maintenance of carbohydrate processing and preservation of TCA cycle activity, thereby strengthening the overall physiological tolerance of ryegrass to heavy metal stress.

## 4. Discussion

### 4.1. Effects of AM Fungi and Cadmium Stress on Ryegrass Growth and Physiology: Metabolic Reprogramming as the Macroscopic Manifestation

Cadmium (Cd), a highly toxic heavy metal, normally inhibits plant growth and photosynthetic activity. Low Cd levels (≤5 mg·kg^−1^) did not inhibit ryegrass growth and even exerted slight stimulatory effects (Figure 1), which agrees with the findings of Ding et al. (2014) [28] and Aina et al. (2007) [29]. Such reactions could be related to Cd-induced increases in indole-3-acetic acid (IAA) and hydrogen peroxide (H_2_O_2_), or Cd-stimulated transpiration and stomatal conductance that transiently enhance photosynthetic activity [30]. At high Cd concentrations (≥50 mg·kg^−1^), however, cells suffer severe damage, photosynthesis is impaired, and metabolism malfunctions, greatly inhibiting growth. These responses are consistent with the pronounced changes in metabolic profiles observed across Cd treatments (Figure 4). Moreover, high Cd levels disrupt the balance of reactive oxygen species (ROS), triggering lipid peroxidation (increased MDA levels) [31] and suppressing key antioxidant enzymes. Cd^2+^ has ionic properties similar to those of essential cations such as Ca^2+^ and Mg^2+^; excessive Cd can displace these ions from enzyme catalytic centers or bind to thiol groups, causing widespread metabolic dysregulation [7,32]. High Cd exposure can also impair chlorophyll biosynthesis and chloroplast structure [33], leading to a significant decline in soluble sugar content (Figure 2).

AM fungal inoculation greatly boosted ryegrass growth at all Cd concentrations, with stronger effects under higher Cd levels. This advantage is attributed to arbuscular mycorrhizal (AM) symbiosis. Specifically, the extraradical hyphae of AMF extend the effective nutrient uptake zone of plants [34], facilitating the acquisition of phosphorus (P), nitrogen (N), and other essential nutrients, thereby alleviating Cd-induced nutritional imbalances [35]. Although AM symbiosis enhanced total Cd uptake in ryegrass roots, most Cd was sequestered in root tissues [36]. This root-specific Cd retention attenuates Cd translocation to aboveground shoots, thereby mitigating Cd-induced damage to photosynthetic tissues [37,38]. Despite a marked decline in AM fungal colonization rates under high Cd stress (Table 2), the fungi nonetheless conferred substantial protective benefits to ryegrass.

AM fungi enhanced ryegrass tolerance to Cd via a multifaceted physiological strategy centered on activation of the antioxidant defense system, maintenance of chlorophyll content, and osmotic adjustment. AMF-inoculated plants exhibited increased activities of catalase (CAT), peroxidase (POD), and superoxide dismutase (SOD), coupled with significantly reduced MDA levels (Figure 2), indicating enhanced ROS scavenging [39]. Improved leaf nutritional status, particularly increased Mg and N contents, likely promoted chlorophyll accumulation by 6.08–15.91% and strengthened the photosynthetic robustness of AMF-inoculated ryegrass. Furthermore, soluble sugar concentrations increased by 5.92–15.97% across Cd treatments in AMF-inoculated plants, contributing to osmotic balance and energy supply under stress [40]. Importantly, these physiological improvements suggest intrinsic metabolic reprogramming in AMF-inoculated ryegrass. For example, higher chlorophyll levels are consistent with activated starch and sucrose metabolism, while enhanced antioxidant enzyme activities correspond to the accumulation of amino acids (e.g., L-alanine, L-aspartate) and phenolic compounds with antioxidative properties. Collectively, these findings indicate that AM fungi improve Cd tolerance mainly by reshaping root–leaf metabolic networks. Since roots are the primary site of fungal colonization and Cd entry, they likely play a central regulatory role [41].

### 4.2. Organ-Specific Metabolic Responses of Ryegrass Under Cd Stress: The Interplay Between Exposure Intensity and Functional Differentiation

Cd stress was associated with apparent organ-specific metabolic changes in ryegrass, which can be attributed to differences in Cd exposure levels and the distinct functional properties of roots and leaves. AM fungi mainly function by restoring or enhancing stress-related metabolites; however, the core pathways involved and the regulatory intensity differ between organs.

Roots, as the primary site of Cd contact, initiated metabolic responses even under low Cd conditions (≤5 mg·kg^−1^). Key stress-defense metabolites (e.g., carbamic acid and 2-propenoic acid) were notably upregulated, and D-galactose levels in the galactose metabolism pathway began to decline. In contrast, leaves exhibited minimal metabolic disruption at low Cd concentrations due to limited Cd translocation, as root Cd levels were approximately 10–12 times higher than those in leaves. Only under high Cd exposure (≥100 mg·kg^−1^) did leaf Cd content increase sharply (e.g., 124.2 mg·kg^−1^ at the 200 mg·kg^−1^ treatment), triggering extensive metabolic suppression, including decreases in citric acid and L-aspartate, with 85.4% of differential metabolites showing reduced abundance [42]. These findings indicate that leaf metabolic responses are delayed relative to those of roots.

Organ-specific metabolic pools drive this directional divergence, with roots and leaves inherently harboring distinct metabolite reservoirs derived from their functional specialization. Notably, host plant metabolites play critical roles in initiating and maintaining symbiosis between plants and AM fungi [43]. Acting as the first line of defense against Cd toxicity, plant roots possess a metabolite pool characterized by a “stress-protective” profile. This pool is enriched in phenolics (e.g., 4-hydroxybenzoic acid and syringic acid) and amines (e.g., dopamine and putrescine), compounds known for strong antioxidant activity and cytoprotective properties [44]. In addition, roots contain substantial carbohydrate reserves (e.g., D-glucose and sucrose) that help maintain cellular osmotic balance and mitigate Cd-induced cellular damage [45]. Under low Cd stress, these metabolites were rapidly mobilized, enabling an effective “frontline defense” mechanism in roots. With increasing Cd levels, however, the activity of key biosynthetic enzymes was suppressed, leading to extensive downregulation of 75.76% of differentially expressed metabolites.

In contrast, metabolic pools in leaves are predominantly composed of metabolites supporting photosynthetic processes, particularly alcohols (e.g., 1-octacosanol and phytol) [46] and organic acids (e.g., citric acid and glyceric acid) [47]. At low Cd exposure, modest increases in organic acid concentrations suggest early detoxification via Cd chelation and reduced Cd bioavailability [48]. High Cd stress disrupted chloroplast integrity and photosynthetic metabolism, suppressing the synthesis of primary photosynthates (e.g., sedoheptulose and D-glucitol) and limiting the accumulation of protective metabolites. Consequently, leaves exhibited a more severe metabolic decline than roots under high Cd stress.

Overall, these results demonstrate that focusing solely on leaf responses would overlook root-driven early defense mechanisms and Cd sequestration. Therefore, an integrated root–leaf framework is essential for a comprehensive understanding of plant Cd tolerance mechanisms.

### 4.3. Differential Modulation of Root and Leaf Metabolism by AM Fungi Under Cd and Non-Cd Conditions

AM fungi significantly influenced ryegrass metabolism regardless of Cd presence, yet the regulatory patterns and intensities were markedly different. In Cd-free soil, AM fungi exerted stronger metabolic impacts on roots than on leaves: 16.47% of root metabolites were altered compared with only 6.67% in leaves. Root metabolites associated with cellular protection (e.g., putrescine) and energy supply (e.g., D-mannose) were elevated [49,50], while sterols and organic acids showed moderate declines. In leaves, only a few metabolites (e.g., pyroglutamic acid and D-mannitol) increased. These patterns reflect the inherent root-centered colonization strategy of AM fungi and their role in optimizing belowground metabolism even in the absence of stress. Under Cd exposure, AM fungi shifted from baseline optimization to precision metabolic repair, displaying distinct organ-specific targeting.

Roots: Galactose metabolism restoration was coupled with the revival of glyoxylate–dicarboxylate metabolism. Notably, high Cd exposure significantly diminished D-galactose concentrations and tricarboxylic acid cycle-related organic acids (e.g., succinate and citrate). AM fungal inoculation reinstated D-galactose levels and increased succinate and citrate concentrations (with respective increases of 27.8% and 31.5%), thereby restoring cellular energy supply and strengthening Cd-chelating capacity via organic acid biosynthesis.

Leaves: Enhancement of starch and sucrose metabolism and amino acid biosynthesis. In leaves exposed to high Cd concentrations, sucrose content and L-alanine levels underwent a marked reduction. Notably, AM fungal inoculation mitigated these declines, restoring sucrose by 22.7–29.4% and enhancing L-alanine content by 31.8% compared to high Cd-stressed plants without AM fungi. These metabolic adjustments facilitate energy supply for photosynthetic repair processes while enhancing the biosynthesis of stress-alleviating amino acids and phenolic compounds.

Cross-organ coordination: AM fungi increased the abundance of amino acids (e.g., L-threonine) and organic acids (e.g., fumarate) in both organs. Enhanced sucrose accumulation in leaves facilitated transport to roots, supplying substrates for stress metabolism, while increased root organic acid synthesis reduced Cd translocation by chelating Cd^2+^ locally. This coordinated response supports the conceptual model of “root detoxification–leaf protection synergy”.

### 4.4. Metabolomics-Based Insights into AM-Enhanced Cd Tolerance

KEGG enrichment analysis revealed distinct organ specificity in the regulatory effects of arbuscular mycorrhizal (AM) fungi on metabolic pathways, highlighting coordinated metabolic adjustments between roots and leaves. Among all enriched pathways, galactose metabolism was identified as a shared pathway across both organs and exhibited significant enrichment (*p* ≤ 0.01) in roots and leaves. Cadmium stress markedly inhibited this pathway, as evidenced by decreased levels of D-galactose and galactinol. Following AM fungal colonization, endogenous levels of these metabolites were restored, thereby facilitating osmotic regulation, carbohydrate turnover, and the provision of precursors for secondary metabolite biosynthesis [51]. The concurrent recovery of galactose metabolism in both roots and leaves emphasizes its role as a core inter-organ metabolic hub coordinating plant-wide responses to Cd stress.

In roots, glyoxylate–dicarboxylate metabolism, an essential branch of the tricarboxylic acid (TCA) cycle, was significantly affected by Cd stress and AM colonization. Cadmium exposure suppressed this pathway, leading to reduced concentrations of key TCA intermediates such as malate and succinate [52], indicating impaired mitochondrial energy metabolism. AM fungal colonization reversed this suppression by restoring succinate and citrate levels, thereby preserving TCA cycle integrity and sustaining cellular energy supply [53]. In addition to their role in energy metabolism, organic acids produced through this pathway contribute directly to Cd detoxification by chelating Cd^2+^ ions, reducing their mobility within root tissues and protecting mitochondrial function [54]. These results are consistent with previous reports demonstrating AM-mediated enhancement of TCA cycle activity in host plants.

In leaves, AM fungal colonization significantly upregulated multiple metabolic pathways associated with photosynthetic maintenance and stress defense. Notably, starch and sucrose metabolism, together with alanine, aspartate, and glutamate metabolism, showed marked enrichment. The accumulation of sucrose and starch supports photosynthetic repair processes and facilitates carbohydrate allocation from leaves to roots under Cd stress. Simultaneously, activation of amino acid metabolic pathways increases the availability of precursors for proline biosynthesis, resulting in its accumulation in leaves. Proline acts as a key osmoprotectant and antioxidant, stabilizing protein structures and scavenging reactive oxygen species (ROS), thereby protecting photosynthetic machinery from Cd-induced oxidative damage [55].

Collectively, these results indicate that AM fungi enhance Cd tolerance through a coordinated, organ-specific metabolic strategy. Roots primarily contribute to Cd immobilization and energy maintenance via organic acid metabolism, whereas leaves focus on sustaining photosynthetic function and producing stress-protective metabolites. The shared restoration of galactose metabolism links these organ-specific responses into an integrated metabolic network, underpinning a whole-plant tolerance mechanism to Cd stress.

## 5. Conclusions

This study demonstrates that arbuscular mycorrhizal fungi (AMF) markedly enhance cadmium (Cd) tolerance in perennial ryegrass. The protective effects were most pronounced under severe Cd stress (soil Cd ≥ 50 mg·kg^−1^), where plant growth was otherwise strongly inhibited. Although AMF colonization levels declined under high Cd exposure, the symbiosis remained functionally effective, indicating that AMF-mediated stress mitigation does not solely depend on colonization intensity, but rather on the coordinated regulation of host physiological and metabolic processes.

At the whole-plant level, AMF confer Cd tolerance through an integrated mechanism encompassing growth maintenance, metabolic reprogramming, and internal Cd immobilization. AMF inoculation significantly improved plant growth and physiological performance under Cd stress by enhancing antioxidant defense systems (e.g., CAT and POD activities), reducing lipid peroxidation (lower MDA levels), and sustaining chlorophyll content, thereby preserving photosynthetic capacity. These physiological benefits provide the foundation for downstream metabolic regulation and detoxification responses.

At the metabolic level, AMF orchestrate coordinated root–leaf metabolic reprogramming in response to Cd stress. In roots, AMF specifically restored Cd-suppressed galactose metabolism and glyoxylate–dicarboxylate pathways, as evidenced by the recovery of D-galactose and key TCA cycle intermediates (succinate and citrate). This metabolic repair helps sustain energy supply and enhances organic acid-mediated Cd chelation, reinforcing the role of roots as the primary detoxification site. In contrast, leaves primarily responded through the reactivation of starch and sucrose metabolism and alanine–aspartate–glutamate pathways, supporting photosynthetic recovery and increasing the production of stress-protective metabolites, including amino acids and other stress-related compounds. Across both organs, the accumulation of stress-related amino acids (e.g., L-threonine) and organic acids (e.g., fumarate) further contributed to metabolic resilience under Cd stress.

At the level of metal partitioning, AMF reshaped internal Cd distribution by promoting root retention and restricting root-to-shoot translocation. The increased root Cd bioconcentration factor and reduced translocation factor effectively limited Cd accumulation in aboveground tissues, thereby protecting photosynthetically active organs from Cd toxicity.

Collectively, these findings support a root-centered detoxification and leaf-protection synergy model, in which AMF enhance Cd tolerance by coupling metabolic repair, energy stabilization, and Cd immobilization in roots with photosynthetic maintenance and stress buffering in leaves. Compared with the relatively modest metabolic adjustments observed under non-stress conditions, AMF under Cd stress triggered a coordinated, multi-level regulatory network integrating physiological performance, metabolic homeostasis, and metal partitioning. This mechanistic framework provides a theoretical basis for applying AMF–plant symbioses in the remediation of moderately to highly Cd-contaminated soils.

## Figures and Tables

**Table 1:** Effects of arbuscular mycorrhizal (AM) fungal inoculation on cadmium (Cd) uptake, accumulation, and translocation from roots to shoots.

Cd^2+^ Content (mg·kg^−1^)	AM Fungal Levels	Cd Content (mg·kg^−1^)		Translocation Factor (TF)	Bioconcentration Factor (BCF)	
		Leaf	Root		Overground	Underground
0	NM	0.07 ± 0.00 ^g^	0.73 ± 0.16 ^i^	0.1 ± 0.02 ^e^	0.37 ± 0.01 ^f^	3.67 ± 0.80 ^f^
	AM	0.09 ± 0.00 ^g^	1.32 ± 0.14 ^i^	0.07 ± 0.01 ^f^	0.46 ± 0.01 ^e^	6.58 ± 0.69 ^c^
5	NM	3.41 ± 0.25 ^g^	37.18 ± 2.22 ^h^	0.09 ± 0.01 ^e^	0.68 ± 0.05 ^cd^	7.44 ± 0.44 ^b^
	AM	4.26 ± 0.51 ^g^	66.57 ± 2.22 ^g^	0.06 ± 0.01 ^f^	0.85 ± 0.10 ^a^	13.31 ± 0.44 ^a^
50	NM	31.90 ± 0.72 ^f^	133.14 ± 7.54 ^f^	0.24 ± 0.01 ^a^	0.64 ± 0.01 ^d^	2.66 ± 0.15 ^g^
	AM	41.05 ± 1.92 ^e^	218.78 ± 2.10 ^e^	0.19 ± 0.01 ^c^	0.82 ± 0.04 ^a^	4.38 ± 0.04 ^e^
100	NM	63.28 ± 0.60 ^d^	381.54 ± 6.42 ^d^	0.17 ± 0.01 ^d^	0.63 ± 0.01 ^d^	3.82 ± 0.06 ^ef^
	AM	79.60 ± 3.87 ^c^	518.58 ± 1.75 ^c^	0.15 ± 0.01 ^d^	0.80 ± 0.04 ^ab^	5.19 ± 0.02 ^d^
200	NM	124.24 ± 1.66 ^b^	530.53 ± 1.89 ^b^	0.23 ± 0.00 ^b^	0.62 ± 0.01 ^d^	2.66 ± 0.15 ^g^
	AM	148.69 ± 5.73 ^a^	718.76 ± 0.32 ^a^	0.21 ± 0.01 ^a^	0.74 ± 0.03 ^bc^	3.59 ± 0.00 ^f^
Cd		1783.283 *** ^(1)^	1715.38 **	229.04 ***	283.01 ***	64.38 ***
AM fungal		194.03 ***	261.05 ***	5.01 ns	102.34 ***	33.02 ***
Cd * AM fungal		10.693 ***	11.91 ns	0.42 ns	4.87 ***	4.38 ***

Note: NM represents the non-mycorrhizal (non-inoculated) treatment, and AM represents the arbuscular mycorrhizal (AM) fungal inoculation treatment. Data are presented as mean ± standard deviation (mean ± SD), with a sample size of *n* = 5. Different superscript letters within the same column indicate significant differences among treatments (*p* < 0.05). ^(1)^ The last three rows of the table show the F-values for the effects of Cd treatment, AM fungal inoculation, and their interaction. * *p* < 0.05, ** *p* < 0.01, *** *p* < 0.001, and ns indicates no significant difference.

**Table 2:** Effects of AM fungi on mycorrhizal colonization of ryegrass under Cd stress.

Cadmium Concentration (mg·kg^−1^)	Mycorrhizal Colonization Rate	
	NM	AM
0	0	56.71 ± 6.69% ^a^
5	0	52.63 ± 2.73% ^a^
50	0	42.68 ± 8.20% ^ab^
100	0	39.79 ± 5.32% ^bc^
200	0	28.91 ± 1.98% ^c^

Note: NM represents the non-mycorrhizal (non-inoculated) treatment, and AM represents the arbuscular mycorrhizal (AM) fungal inoculation treatment. Different superscript letters within the same column indicate significant differences among treatments (*p* < 0.05).

## Data Availability

The raw data supporting the conclusions of this article will be made available by the authors on request.

## References

[1] Wieczorek J., Baran A., Bubak A. (2023). Mobility, Bioaccumulation in Plants, and Risk Assessment of Metals in Soils. Sci. Total Environ..

[2] Luo J., Feng S., Ning W., Liu Q., Cao M. (2025). Integrated Source Analysis and Network Ecological Risk Assessment of Soil Heavy Metals in Qinghai–Tibet Plateau Pastoral Regions. J. Hazard. Mater..

[3] Xia F., Zhao Z., Niu X., Wang Z. (2024). Integrated Pollution Analysis, Pollution Area Identification and Source Apportionment of Heavy Metal Contamination in Agricultural Soil. J. Hazard. Mater..

[4] Zha X., An J., Deng L., Gao X., Tian Y. (2024). Risk Assessment and Source Apportionment of Heavy Metals in the Soil–Water-Grain System in a Typical Area of the Central Qinghai–Tibet Plateau. Ecol. Indic..

[5] Kubier A., Wilkin R.T., Pichler T. (2019). Cadmium in Soils and Groundwater: A Review. Appl. Geochem..

[6] Shang E., Long A., Yang J., Ma Y., Yao W., Zhang S. (2025). Dynamics of Cadmium Pollution Risk in Agricultural Land Soil of Tropical Islands in China from 2000 to 2024: A Case Study of Hainan Island. Appl. Sci..

[7] Yang Y., Hassan M.F., Ali W., Zou H., Liu Z., Ma Y. (2025). Effects of Cadmium Pollution on Human Health: A Narrative Review. Atmosphere.

[8] Zhuang X., Liu S., Xu S., Qin S., Lyu D., He J., Zhou J. (2025). Arbuscular Mycorrhizal Fungi Alleviate Cadmium Phytotoxicity by Regulating Cadmium Mobility, Physiological Responses, and Gene Expression Patterns in *Malus hupehensis* Rehd. Int. J. Mol. Sci..

[9] Zhou Y., Jin Z., Ren X., Hong C., Hua Z., Zhu Y., Dong Y., Li X. (2024). Symbiotic Conserved Arbuscular Mycorrhiza Fungi Supports Plant Health. Sci. Total Environ..

[10] Zárate Martínez O., Hiiesalu I., Sepp S.-K., Koorem K., Vasar M., Wipulasena A.Y.A.P., Liu S., Astover A., Öpik M., Pärtel M. (2024). Arbuscular Mycorrhizal Fungal Diversity in Agricultural Fields Is Explained by the Historical Proximity to Natural Habitats. Soil Biol. Biochem..

[11] Zhao S., Yan L., Kamran M., Liu S., Riaz M. (2024). Arbuscular Mycorrhizal Fungi-Assisted Phytoremediation: A Promising Strategy for Cadmium-Contaminated Soils. Plants.

[12] Zhang D., Liu X., Zhang Y., Ye J., Yi Q. (2025). Effects of Arbuscular Mycorrhizal Fungi on the Physiological Responses and Root Organic Acid Secretion of Tomato (*Solanum lycopersicum*) Under Cadmium Stress. Horticulturae.

[13] Peng Z., Xing Y., Ma Y., Li S., Jia Y., Yang H., Zhang F. (2025). Arbuscular Mycorrhizal Fungi Enhance Soybean Phosphorus Uptake and Soil Fertility under Saline-Alkaline Stress. Sci. Rep..

[14] Lan Z., He Q., Zhang M., Liu H., Fang L., Nie J. (2023). Assessing the Effects of Cadmium Stress on the Growth, Physiological Characteristics, and Metabolic Profiling of Rice (*Oryza sativa* L.) Using HPLC-QTOF/MS. Chemosensors.

[15] Liao S., Ling Y., Gao Y., Ma G., Li X., Chen L., Hu L., Xie Y. (2025). Enhanced Cadmium Tolerance in Perennial Ryegrass via Exogenous Application of *Enterobacter hormaechei* Strain X20. Ecotoxicol. Environ. Saf..

[16] Yang W., Liu F., Wu G., Liang S., Bai X., Liu B., Zhang B., Chen H., Yang J. (2024). Widely Targeted Metabolomics Analysis of the Roots, Stems, Leaves, Flowers, and Fruits of *Camellia luteoflora*, a Species with an Extremely Small Population. Molecules.

[17] Wu X., Li X., Wang W., Shan Y., Wang C., Zhu M., La Q., Zhong Y., Xu Y., Nan P. (2020). Integrated Metabolomics and Transcriptomics Study of Traditional Herb *Astragalus membranaceus* Bge. Var. Mongolicus (Bge.) Hsiao Reveals Global Metabolic Profile and Novel Phytochemical Ingredients. BMC Genom..

[18] Nakabayashi R., Hashimoto K., Toyooka K., Saito K. (2017). Top-down Metabolomic Approaches for Nitrogen-Containing Metabolites. Anal. Chem..

[19] Wu Y., Huang X., Yang H., Zhang S., Lyu L., Li W., Wu W. (2023). Analysis of Flavonoid-Related Metabolites in Different Tissues and Fruit Developmental Stages of Blackberry Based on Metabolome Analysis. Food Res. Int..

[20] Janeeshma E., Puthur J.T., Wróbel J., Kalaji H.M. (2022). Metabolic Alterations Elicited by Cd and Zn Toxicity in *Zea mays* with the Association of *Claroideoglomus claroideum*. Ecotoxicology.

[21] Wang J., Chen X., Chu S., Hayat K., Chi Y., Liao X., Zhang H., Xie Y., Zhou P., Zhang D. (2024). Conjoint Analysis of Physio-Biochemical, Transcriptomic, and Metabolomic Reveals the Response Characteristics of *Solanum nigrum* L. to Cadmium Stress. BMC Plant Biol..

[22] Feng Z., Liu N., Tu P., Zou Y., Vosatka M., Zhao Z., Chen J., Song H. (2024). Metabolomics Analysis of Bahia Grass (*Paspalum notatum*) Inoculated with Arbuscular Mycorrhizal Fungi Exposed to Soil Cd Stress. Environ. Exp. Bot..

[23] Dai H., Xiao C., Liu H., Hao F., Tang H. (2010). Combined NMR and LC-DAD-MS Analysis Reveals Comprehensive Metabonomic Variations for Three Phenotypic Cultivars of *Salvia miltiorrhiza* Bunge. J. Proteome Res..

[24] Zhang X.-F., Hu Z.-H., Yan T.-X., Lu R.-R., Peng C.-L., Li S.-S., Jing Y.-X. (2019). Arbuscular Mycorrhizal Fungi Alleviate Cd Phytotoxicity by Altering Cd Subcellular Distribution and Chemical Forms in *Zea mays*. Ecotoxicol. Environ. Saf..

[25] Jensen H., Lehto N., Almond P., Gaw S., Robinson B. (2023). The Uptake of Rare Trace Elements by Perennial Ryegrass (*Lolium perenne* L.). Toxics.

[26] Brunelli C., Bicchi C., Di Stilo A., Salomone A., Vincenti M. (2006). High-Speed Gas Chromatography in Doping Control: Fast-GC and Fast-GC/MS Determination of Beta-Adrenoceptor Ligands and Diuretics. J. Sep. Sci..

[27] Delpiano C.A., Rios R.S., Barraza-Zepeda C.E., Pozo M.J., Aguilera L.E., Loayza A.P. (2024). Arbuscular Mycorrhizal Colonization Defines Root Ecological Strategies in an Extreme Arid Environment. Front. Plant Sci..

[28] Ding Y., Feng R., Wang R., Guo J., Zheng X. (2014). A Dual Effect of Se on Cd Toxicity: Evidence from Plant Growth, Root Morphology and Responses of the Antioxidative Systems of Paddy Rice. Plant Soil.

[29] Aina R., Labra M., Fumagalli P., Vannini C., Marsoni M., Cucchi U., Bracale M., Sgorbati S., Citterio S. (2007). Thiol-Peptide Level and Proteomic Changes in Response to Cadmium Toxicity in *Oryza sativa* L. Roots. Environ. Exp. Bot..

[30] Delpérée C., Lutts S. (2008). Growth Inhibition Occurs Independently of Cell Mortality in Tomato (*Solanum lycopersicum*) Exposed to High Cadmium Concentrations. J. Integr. Plant Biol..

[31] Lee J.-Y., Tokumoto M., Satoh M. (2025). Molecular Mechanisms of Cadmium-Induced Toxicity and Its Modification. Int. J. Mol. Sci..

[32] Sanità di Toppi L., Gabbrielli R. (1999). Response to Cadmium in Higher Plants. Environ. Exp. Bot..

[33] Zhang H., Xu Z., Huo Y., Guo K., Wang Y., He G., Sun H., Li M., Li X., Xu N. (2020). Overexpression of *Trx CDSP32* Gene Promotes Chlorophyll Synthesis and Photosynthetic Electron Transfer and Alleviates Cadmium-Induced Photoinhibition of PSII and PSI in Tobacco Leaves. J. Hazard. Mater..

[34] Campos C., Carvalho M., Brígido C., Goss M.J., Nobre T. (2018). Symbiosis Specificity of the Preceding Host Plant Can Dominate but Not Obliterate the Association Between *Wheat* and Its Arbuscular Mycorrhizal Fungal Partners. Front. Microbiol..

[35] Shen K., He Y., Xia T., Guo Y., Wu B., Han X., Chen H., Zhao Y., Li J., Gao L. (2023). Arbuscular Mycorrhizal Fungi Promote Superior Root Trait Combinations Conducive to Soil Nutrient Acquisition by Natives Relative to Invaders. Rhizosphere.

[36] Sun S., Fan X., Feng Y., Wang X., Gao H., Song F. (2023). Arbuscular Mycorrhizal Fungi Influence the Uptake of Cadmium in Industrial Hemp (*Cannabis sativa* L.). Chemosphere.

[37] Kuang Q., Wu Y., Gao Y., An T., Liu S., Liang L., Xu B., Zhang S., Yu M., Shabala S. (2025). Arbuscular Mycorrhizal Fungi Mitigate Cadmium Stress in Maize. Ecotoxicol. Environ. Saf..

[38] Chen S., Jin W., Liu A., Zhang S., Liu D., Wang F., Lin X., He C. (2013). Arbuscular Mycorrhizal Fungi (AMF) Increase Growth and Secondary Metabolism in Cucumber Subjected to Low Temperature Stress. Sci. Hortic..

[39] Azcón R., Perálvarez M.D.C., Biró B., Roldán A., Ruíz-Lozano J.M. (2009). Antioxidant Activities and Metal Acquisition in Mycorrhizal Plants Growing in a Heavy-Metal Multicontaminated Soil Amended with Treated Lignocellulosic Agrowaste. Appl. Soil Ecol..

[40] Hai X., Mi J., Zhao B., Zhang B., Zhao Z., Liu J. (2022). Foliar Application of Spermidine Reduced the Negative Effects of Salt Stress on Oat Seedlings. Front. Plant Sci..

[41] Chen J., Wang L., Liang X., Li B., He Y., Zhan F. (2023). An Arbuscular Mycorrhizal Fungus Differentially Regulates Root Traits and Cadmium Uptake in Two Maize Varieties. Ecotoxicol. Environ. Saf..

[42] Yannarelli G.G., Fernández-Alvarez A.J., Santa-Cruz D.M., Tomaro M.L. (2007). Glutathione Reductase Activity and Isoforms in Leaves and Roots of Wheat Plants Subjected to Cadmium Stress. Phytochemistry.

[43] Kaur S., Suseela V. (2020). Unraveling Arbuscular Mycorrhiza-Induced Changes in Plant Primary and Secondary Metabolome. Metabolites.

[44] Wang Y., Xu L., Shen H., Wang J., Liu W., Zhu X., Wang R., Sun X., Liu L. (2015). Metabolomic Analysis with GC-MS to Reveal Potential Metabolites and Biological Pathways Involved in Pb & Cd Stress Response of *Radish* Roots. Sci. Rep..

[45] Long R.W., Adams H.D. (2023). The Osmotic Balancing Act: When Sugars Matter for More than Metabolism in Woody Plants. Glob. Change Biol..

[46] Dörmann P., Benning C. (2002). Galactolipids Rule in Seed Plants. Trends Plant Sci..

[47] Robinson S.P. (1984). Lack of ATP Requirement for Light Stimulation of Glycerate Transport into Intact Isolated Chloroplasts. Plant Physiol..

[48] Allan J., Cameron J., Bruno J. (2022). A Systematic Review of Recreational Nitrous Oxide Use: Implications for Policy, Service Delivery and Individuals. Int. J. Environ. Res. Public Health.

[49] Kuznetsov V., Shorina M., Aronova E., Stetsenko L., Rakitin V., Shevyakova N. (2007). NaCl- and Ethylene-Dependent Cadaverine Accumulation and Its Possible Protective Role in the Adaptation of the Common Ice Plant to Salt Stress. Plant Sci..

[50] Harris G.C., Gibbs P.B., Ludwig G., Un A., Sprengnether M., Kolodny N. (1986). Mannose Metabolism in Corn and Its Impact on Leaf Metabolites, Photosynthetic Gas Exchange, and Chlorophyll Fluorescence. Plant Physiol..

[51] Guo S., Song J., Zhang B., Jiang H., Ma R., Yu M. (2018). Genome-Wide Identification and Expression Analysis of Beta-Galactosidase Family Members during Fruit Softening of Peach [*Prunus persica* (L.) Batsch]. Postharvest Biol. Technol..

[52] He L., Jing Y., Shen J., Li X., Liu H., Geng Z., Wang M., Li Y., Chen D., Gao J. (2019). Mitochondrial Pyruvate Carriers Prevent Cadmium Toxicity by Sustaining the TCA Cycle and Glutathione Synthesis. Plant Physiol..

[53] Schweiger R., Baier M.C., Persicke M., Müller C. (2014). High Specificity in Plant Leaf Metabolic Responses to Arbuscular Mycorrhiza. Nat. Commun..

[54] Pampolino M.F., Laureles E.V., Gines H.C., Buresh R.J. (2008). Soil Carbon and Nitrogen Changes in Long-Term Continuous Lowland Rice Cropping. Soil Sci. Soc. Am. J..

[55] Verma P., Mathur A.K., Shanker K. (2012). Increased Availability of Tryptophan in 5-Methyltryptophan-Tolerant Shoots of *Catharanthus roseus* and Their Postharvest in Vivo Elicitation Induces Enhanced Vindoline Production. Appl. Biochem. Biotechnol..

